# Clinical characteristics and treatment outcomes of patients with insulinoma–a single center's experience of 76 cases over a 10-year period

**DOI:** 10.1016/j.heliyon.2025.e41799

**Published:** 2025-01-10

**Authors:** Wei Li, Yali Cheng, Qingyong Ma, Zheng Wu, Zheng Wang

**Affiliations:** Department of Hepatobiliary Surgery, The First Affiliated Hospital of Xi'an Jiaotong University, Xi'an, Shaanxi 710061, PR China

**Keywords:** Insulinoma, Pancreatic neuroendocrine neoplasm, Surgery, Pancreatic head/neck tumor

## Abstract

**Objective:**

Insulinoma is a rare pancreatic neuroendocrine neoplasm caused by pancreatic beta cell tumor or beta cell proliferation resulting in excessive insulin secretion. Due to its rarity, surgical analysis and summary studies on patients with insulinoma are few and the sample size is limited.

**Methods:**

In this study, we retrospective the cumulative experiences including demographics, clinical characteristics, detailed surgical reports and postoperative outcome of 76 patients diagnosed with insulinoma from 2010 to 2020 at the First Affiliated Hospital of Xi'an Jiaotong University.

**Results:**

Our cohort consisted of 48 females and 28 males with an average diagnosis age of 52 years. Loss of consciousness (68 %), sweating (59 %), vertigo (56 %) are three most shown symptoms. The average time from symptom onset to diagnosis of insulinoma was 33.4 month. When CT combined with MRI was used, the diagnostic rate was as high as 96.87 %. Of the 76 patients, 48.68 % of preoperative tumors were in the head/neck, and the remaining 51.32 % were in the body/tail. Most of the patients received surgery for enucleation (65.79 %). The duration of surgery was 216 ± 105 min. The incidence of postoperative pancreatic fistula was 53.95 %. Postoperative pancreatic hemorrhage occurred in 6 patients (7.89 %), all of whom were pancreatic head/neck surgery patients. The incidence of pancreatic fistula, bleeding and major complications after the operation of pancreatic head/neck insulinoma was higher than that of pancreatic body/tail insulinoma.

**Conclusion:**

CT combined with MRI can localize insulinoma in most patients. As a parenchyma-sparing pancreatectomy, enucleation is the procedure of choice if possible. The incidence of postoperative hemorrhage should be more vigilant after the surgery of pancreatic head/neck insulinoma. The intraoperative suture of high-risk vessels prone to bleeding should be performed if necessary.

## 1 Introduction

Pancreatic neuroendocrine neoplasms (PNENs) are a rare class of tumors that are classified into functional and non-functional according to their clinical manifestations [1]. Among them, insulinoma is the most common functional PNEN that is characterized by excessive amounts of insulin secreted. Typically, these patients present with the Whipple's triad, which includes hypoglycemic symptoms, hypoglycemia (blood sugar levels ≤50 mg/dL), and symptoms relief after glucose administration [2]. Despite the classical symptoms, diagnosis of these tumors are often delayed in clinical practice [3]. Preoperative diagnosis of insulinoma is often challenging. Tumors distributed throughout the pancreatic gland are usually small. Thus, the tumors are difficult to detect by routine computed tomography (CT) [4]. However, the combination of cross-sectional imaging techniques, with endoscopic ultrasonography (EUS) and nuclear medicine, has dramatically improved the preoperative assessment of insulinomas [5]. EUS is very suitable for close-up imaging of pancreatic lesions. In addition, EUS can achieve insulinoma fine needle puncture [6] and define grading in patients with PNENs [7]. Surgical treatments for insulinoma mainly include Enucleation, Pancreaticoduodenectomy, Duodenum-preserving pancreatic head resection, Middle segment pancreatectomy, Distal pancreatectomy and Distal pancreatectomy with splenectomy [8]. The surgical management or the choice of a less invasive treatment relies to the tumor grade [7]. However, the impact of tumor location on patient survival in insulinoma remains controversial.

The primary aim of this study was to report the surgical experience on insulinoma of 76 insulinoma patients at the first affiliated hospital of Xi'an Jiaotong University in China between 2010 and 2020. Secondary aims were to compare the clinical characteristics of pancreatic head/neck tumor and body/tail tumor in 76 insulinoma patients.

## 2 Patients and methods

**Fig. 1:**
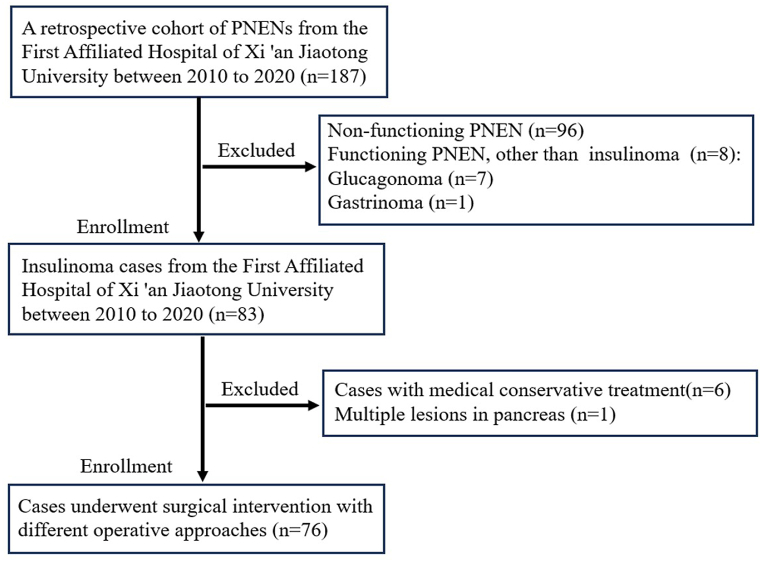
Flow-chart for the inclusion of insulinoma patients in this study. PNENs: Pancreatic neuroendocrine neoplasms.

This is a retrospective case series study conducted at the First Affiliated Hospital of Xi'an Jiaotong University in Xi'an of China. Patients who underwent surgery at the First Affiliated Hospital of Xi 'an Jiaotong University between 2010 and 2020 and histopathological diagnosed with insulinoma were enrolled in this study. Demographic, clinical, radiological, pathologic, and follow-up data were included in the study. Exclusion criteria were: 1)Non-functioning PNEN or functioning PNEN, other than insulinoma,2)multiple lesions in pancreas,3)history of other malignant tumors and severe cardiac or pulmonary disease (Fig. 1).

The study protocol was approved by the Ethics Committee of the First Affiliated Hospital of Xi'an Jiaotong University and the study was conducted in accordance with the approved guidelines. Informed consent was obtained from all subjects and/or their legal guardian(s).

Medical records of these patients were reviewed for data that include demographics, elapsed time between symptoms onset and diagnoses, clinical symptoms at admission, biochemical tests to confirm insulinoma, diagnostic imaging methods and their accuracy, location and size of insulinoma, type of surgery, histopathological examination, postoperative complications for descriptive purposes. Clinically relevant postoperative pancreatic fistula (CR-POPF) was diagnosed and graded according to the International Study Group of Pancreatic Fistula (ISGPF). Grade B and C fistula were regarded as CR-POPF [9]. According to Clavien-Dindo classification, major complications were defined as Clavien-Dindo Grade ≥ III [10]. All the patients were followed up at least 1 year after surgery.

Data were expressed as mean ± standard deviation (SD) and n refers to the number of patients. Unpaired Student's t-test was applied to compare two sets of data. One-way analysis of variance (ANOVA) with Dunnett's post-test was used for multiple comparisons. Data were statistically analyzed using SPSS version 22.0 software (IBM Inc.; Armonk, NY, USA). Statistical significance was set at <0.05. Group differences with continuous data were analyzed using independent samples *t*-test.

## 3 Results

### 3.1 Clinical characteristics of patients with insulinoma

**Fig. 2:**
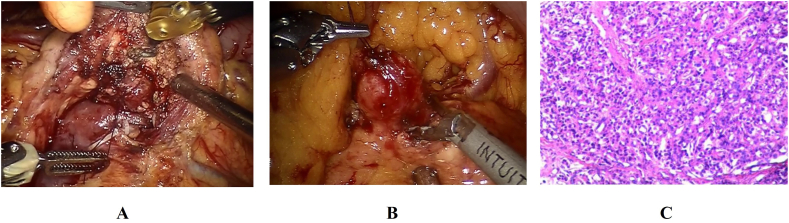
Surgical and pathology images of insulinoma A–B: Surgical photographs of insulinoma enucleation, C: Histopathological examination of insulinoma.

**Fig. 3:**
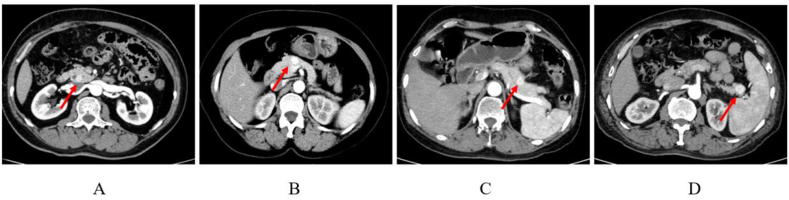
Enhanced computed tomography images of the abdomen. Arrow shows the hyperintense insulinoma in the pancreatic head (A), neck (B), body (C), and tail (D).

The final study population included 76 patients who underwent surgery for pancreatic insulinoma, 48 (63 %) of whom were women and 28 (37 %) were men, with a median age at diagnosis of 52 years (Table 1). The mean time from symptom onset to diagnosis of insulinoma was 33.4 months. The clinical symptoms at admission and the frequency are presented in Table 1. Loss of consciousness (68 %), sweating (59 %), vertigo (56 %) are three most shown symptoms. Other symptoms include weight gain, palpitations, paraphasia, tremor, seizure and sleep disorder. Since insulinoma can take a long time from the onset of symptoms to diagnosis, the above symptoms need to be alert to the presence of insulinoma. For diagnosis, several biochemical tests were performed to confirm insulinoma. All the 76 patients were tested hypoglycemia. Lowest blood glucose level during hospitalization was 1.79 ± 0.56 mmol/L (normal range 3.9–6.1 mmol/L). the Insulin level was 40.32 ± 21.75 μIU/mL (normal range 6–27 μIU/mL). C-peptide was 3.81 ± 2.13 ng/mL (normal range 0.9–4 ng/mL). Histological examination (hematoxylin-eosin staining, Fig. 2) and immunohistochemical results (Table 1) of surgical specimens revealed insulinoma. In the process of imaging examination of insulinoma, the imaging methods and their diagnostic accuracy are also different (Table 1). Enhanced CT images shows the hyperintense insulinoma in the pancreas tissue (Fig. 3). Only almost half of the patients who got Ultrasound have been confirmed insulinoma (49.18 %). The diagnosis rates of CT and MRI were 83.56 % and 78.78 % respectively. However, when CT combined with MRI was used, the diagnostic rate was as high as 96.87 %. 12 patients underwent EUS scanning, with an accuracy diagnostic rate of 83.33 %. Due to financial constraints, we were able to get ^68^Ga DOTA PETs done in 7 patients only and it detected the tumor in 6 patients (85.71 %).

**Table 1:** Characteristics of the study population (n = 76).

Characteristics	Value
Age (years), mean ± SD	52 ± 15
Sex (female)	48 (63 %)
BMI (kg/m^2^), mean ± SD	25.8 ± 4.1
MEN-1 syndrome	3 (3.9 %)
ASA score	
I/II	51 (67 %)
III/IV	25 (33 %)
Time from onset of symptoms to diagnosis (months), mean ± SD	33.4 ± 29.7
Frequency of clinical symptoms at admission	
Loss of consciousness	52 (68 %)
Sweating	45 (59 %)
Vertigo	43 (56 %)
Weight gain	28 (36 %)
Palpitations	27 (35 %)
Paraphasia	23 (30 %)
Tremor	17 (22 %)
Seizure	13 (17 %)
Sleep disorder	13 (17 %)
Biochemical tests to confirm insulinoma	
Hypoglycemia	76 (100 %)
Lowest blood glucose level during hospitalization	1.79 ± 0.56 mmol/L
Insulin level	40.32 ± 21.75μIU/mL
C-peptide:	3.81 ± 2.13 ng/mL
Accuracy of imaging tests for insulinoma detection	
Ultrasound	30/61 (49.18 %)
Computed tomography (CT)	61/73 (83.56 %)
Magnetic resonance imaging (MRI)	26/33 (78.78 %)
CT and MRI	31/32 (96.87 %)
EUS	10/12 (83.33 %)
Somatostatin receptor imaging	6/7 (85.71 %)
Immunohistochemical results	
Synaptophysin	76/76 (100 %)
Chromogranin A	76/76 (100 %)
Insulin	66/76 (86.84 %)
Histology Grade	
G1/G2	44/32

### 3.2 Surgical characteristics of patients with insulinoma

The tumor size was 1.62 ± 0.59 cm (Table 2). All the patients underwent surgeries. 50 of 76 patients received surgery for enucleation (65.79 %, Fig. 2), in which 41 patients (82.00 %) underwent minimally invasive pancreatic tumor enucleation (8 robotic and 33 laparoscopic) and 9 patients (18.00 %) underwent open surgery. 15 of 76 underwent surgery for distal pancreatectomy (19.74 %). The remaining patients received pancreaticoduodenectomy (1 of 76, 1.32 %),Duodenum-preserving pancreatic head resection (6 of 76, 7.89 %),Middle segment pancreatectomy (2 of 76, 2.63 %) and Distal pancreatectomy with splenectomy (2 of 76, 2.63 %). In some cases that tumors closely attached to the main pancreatic duct (12/76, 15.79 %), especially for the pancreatic head/neck tumor, endoscopic pancreatic stents placement was performed.

**Table 2:** Outcome after surgery for 76 insulinoma patients.

Characteristics	Value
Preoperative tumor location	
Head and neck	37 (48.68 %)
Body and tail	39 (51.32 %)
Tumor size (cm)	1.62 ± 0.59
Type of surgery	
Enucleation	50 (65.79 %)
Pancreaticoduodenectomy	1 (1.32 %)
Duodenum-preserving pancreatic head resection	6 (7.89 %)
Middle segment pancreatectomy	2 (2.63 %)
Distal pancreatectomy	15 (19.74)
Distal pancreatectomy with splenectomy	2 (2.63 %)
Performance outcomes	
Duration of surgery (min), mean ± SD	216 ± 105
Blood loss (ml), mean ± SD	151 ± 249
Blood transfusion	1 (1.32 %)
Pancreatic fistula	41 (53.95 %)
Grade B/C	14 (18.42 %)
Hemorrhage	6 (7.89 %)
Delayed gastric emptying	3 (3.95 %)
Length of hospital stay (days), mean ± SD	22 ± 16
Length of stay after operation (days), mean ± SD	12 ± 16
Hospital readmission	3 (3.95 %)
90-day mortality	1 (1.32 %)

The duration of surgery was 216 ± 105 min. The blood loss during the surgery was 151 ± 249 mL. Only one patient received blood transfusion in the operation. After surgery, pancreatic fistula occurred in 41 of 76 patients (53.95 %), grade B/C pancreatic fistula accounted for 18.42 % of the total. Other postoperative complications include hemorrhage (7.89 %) and delayed gastric emptying (3.95 %). There were 6 patients with hemorrhage after pancreatic surgery (7.89 %), all of whom were patients with pancreatic head/neck surgery. 44 of 76 tumors were benign, well-differentiated neuroendocrine tumor G1, while the other 32 tumors were G2. There was no lymph node metastasis in the postoperative pathological results (Table 1). We diagnosed three MEN-I syndrome patients, who also developed with parathyroid tumor or hypophyseal tumor.

The length of hospital stay was 22 ± 16 days, and the length of stay after operation was 12 ± 16 days. The 90-day mortality was 1.32 %, with one death due to ischemic hypoxic encephalopathy caused by hemorrhagic shock.

The median follow-up period was 68.0 months. All patients were cured of the disease with normal glycemia and insulin blood levels. None of the them developed recurrence.

### 3.3 Comparison of clinical characteristics between the pancreatic head/neck tumor and body/tail tumor

Of the 76 patients, preoperative tumors were localized in the head and neck of 37 patients (48.68 %), and in the body and tail of the remaining 39 patients (51.32 %). The clinical characteristics between the pancreatic head/neck tumor and body/tail tumor was shown in Table 3. After operation, there was no difference in basic information (age, sex, BMI and ASA scores) between the two groups. The incidence of grade B/C pancreatic fistula (P = 0.037) and major complications (P = 0.002) after the operation of insulinoma in the head/neck of the pancreas is higher than that of tumors in the body/tail of the pancreas. Among 37 patients who underwent surgery for pancreatic head/neck insulinoma, 9 had major complications, including 6 cases of hemorrhage, 1 case of delayed gastric emptying (DGE), and 2 cases of abdominal infection. In the pancreatic head/neck tumor group, 6 patients (16.22 %) had Grade III complications (3 CR-POPF, intraabdominal bleeding treated by digital subtraction angiography (DSA), 2 CR-POPF, intraabdominal infections treated by drainage, 1 DGE treated by endoscopic nasojejunal feeding tube placement). 2 patients (5.41 %) had Grade IV complications (CR-POPF, intraabdominal bleeding treated by operation, MODS), 1 patient (2.70 %) had Grade V complications (CR-POPF, intraabdominal bleeding treated by operation, hemorrhagic shock, ischemic hypoxic encephalopathy, died in the intensive care unit). In the pancreatic body/tail tumor group, 2 patients (5.13 %) had Grade III complications (intraabdominal infections treated by drainage). We further compare the differences of adverse events in tumor enucleation between the two groups. Among 28 patients who underwent enucleation for pancreatic head/neck insulinoma, 4 (14.29 %) had major complications, including 3 cases of hemorrhage and 1 cases of abdominal infection. In the pancreatic body/tail tumor group (22 patients), only 1 patient (4.55 %) had major complications (intraabdominal infections).

**Table 3:** Comparison of clinical characteristics for pancreatic head/neck tumor and body/tail tumor of 76 insulinoma patients.

Insulinoma		Head/neck (37)	Body/tail (39)	*P*-Value
Age (year)	<52	19	12	0.102
	≥52	18	27	
Sex	Male	13	15	0.815
	Female	24	24	
BMI (kg/m^2^)	<25	14	19	0.364
	≥25	23	20	
ASA scores	I/II	27	24	0.335
	III/IV	10	15	
Time from onset of symptoms to diagnosis (years)	≤2	20	21	1.000
	>2	17	18	
Duration of surgery (min)	≤180	19	21	1.000
	>180	18	18	
Pancreatic fistula	No	16	19	0.037
	Grade A	10	17	
	Grade B/C	11	3	
Complications	No	20	35	0.002
	Grade I–II	8	2	
	Grade III	6	2	
	Grade IV	2	0	
	Grade V	1	0	
Length of stay after operation (days)	<10	16	23	0.251
	≥10	21	16	

The 6 patients with postoperative hemorrhage were all patients with pancreatic head/neck insulinoma surgery. Hemostasis was performed in 1 case by dorsal pancreatic artery suture, 3 cases by gastroduodenal artery (GDA) intervention, and the other 2 cases by anterior superior pancreaticoduodenal artery (ASPDA) suture. This reminds us to alert GDA, ASPDA and dorsal pancreatic artery bleeding after the operation of pancreatic head/neck insulinoma. The intraoperative suture should be performed if necessary.

## 4 Discussion

Insulinoma is a rare disease, and they are the most common type of functional PNETs [11,12]. Most of the previous literature reports were case reports, case series or reviews [1,8,13]. In this study, for the first time, we retrospectively reviewed the surgical strategy information of up to 76 patients with insulinoma for a 10-year period at the First Affiliated Hospital of Xi'an Jiaotong University in China.

Insulinoma have been reported to appear at all ages with a peak of 50's and without any significant gender differences [14]. Our results agree with them showing that the mean age at presentation is 52. In addition, our results are consistent with those of previous studies, suggesting either the same sex distribution or a slight predominance in women (63 %) [15].

The symptoms of insulinoma are non-specific such as loss of consciousness, sweating, vertigo and others include weight gain, palpitations, paraphasia, tremor, seizure and sleep disorder [16]. Failure to recognize these symptoms can lead to a misdiagnosis [15]. Our results showed that in 76 people it took up to 33.4 ± 29.7 months from symptom to diagnosis of insulinoma. Therefore, the above symptoms need to be vigilant about the existence of insulinoma.

Previous studies have reported that insulinoma may be located anywhere in the pancreas but evenly distributed in different parts of the pancreas [17]. Our results are in line with previous data showing no difference in the distribution of insulinoma in the head/neck (48.68 %) and in the body/tail (51.32 %) of the pancreas. Our report showed that the mean tumor size was 1.62 cm. This is consistent with previous reports that insulinoma tend to be less than 2 cm in size, while malignancies are usually more than 3 cm in diameter [18].

In the present study, the diagnostic rates of CT and MRI were 83.56 % and 78.78 %, respectively. However, when CT was combined with MRI, the diagnosis rate was as high as 96.87 %. Therefore, the combination of CT and MRI is a good method for diagnosis of insulinoma. In contrast, ultrasound was less than 50 % positive for insulinoma diagnosis. Due to the low detection rate ultrasound may not be suitable for insulinoma screening. Notably, EUS and somatostatin receptor imaging also showed high accurate diagnostic rate.

The procedure of pancreatic enucleation is commonly performed for insulinoma, with the objective of achieving both radical resection and preservation of the pancreatic parenchyma [19]. Enucleation is recommended for solitary, small (less than 2 cm), superficially located, and clearly defined tumors that are in a favorable position relative to the pancreatic duct. However, tumors that located in the pancreatic head or uncinate process and close proximity to the main pancreatic duct (≤2 mm) have been reported as independent risk factors for POPF after enucleation [20]. In the current study, 65.79 % patients received surgery for enucleation, in which 82.00 % of the patients underwent minimally invasive pancreatic tumor enucleation (8 robotic and 33 laparoscopic). Recent studies have proven that the minimally invasive enucleation for benign sporadic insulinomas is a secure surgical approach that has similar short-term and long-term postoperative outcome compared with the open surgery [21,22].

In those cases that tumors closely attached to the main pancreatic duct (12/76, 15.79 %), endoscopic pancreatic stents placement was performed. There are some advantages for using endoscopic pancreatic stents. It can not only enable clear visualization of the main pancreatic duct through intraoperative ultrasound, facilitating the confirmation of appropriate surgical margin, but also detect the injury of main pancreatic duct injuries visually through the stent color [23].

Recently, endoscopic ultrasound (EUS)-guided ablation therapy and ethanol injection have been reported as a more minimally invasive treatment option for patients with insulinoma [24,25]. The benefits of EUS-guided therapy compared to surgical resection include a shorter Hospital stay, lower incidence of complications and preservation of pancreatic exocrine and endocrine functions [26]. This therapy is also suitable for patients with poor surgical tolerance. The limitations of EUS-guided therapy include some adverse events, such as severe pancreatitis, pancreatic necrosis and perforation. In addition, the safety of ablation treatment for insulinoma in proximity to the main pancreatic duct has not yet been established. Thus, long-term follow up is necessary for proving the efficacy of EUS-guided therapy [27].

We compared the clinical characteristics for pancreatic head/neck tumor and body/tail tumor of insulinoma. Although the impact of tumor location on patient survival for pancreatic cancer has been controversial, previous study has investigated the association between tumor characteristics such as size and stage, and overall survival rates among resectable patients. Patients with pancreatic head cancer had worse overall and disease-free survival than those with pancreatic body/tail cancer. However, it was confirmed that tumor location was not an independent prognostic factor [28]. In the present study, all patients (6 cases) with postoperative hemorrhage were patients with pancreatic head/neck surgery. The incidence of grade B/C pancreatic fistula and major complications after the operation of insulinomas in the head/neck of the pancreas was higher. The bleeding sites were from dorsal pancreatic artery, gastroduodenal artery and anterior superior pancreaticoduodenal artery. This reminds us to alert bleeding in these areas after the operation of pancreatic head/neck insulinoma.

This study has several limitations. Firstly, as a single-center retrospective study, there is a comparatively limited sample size and potential for selection bias. In addition, for a thorough assessment of the long-term outcomes, it is necessary to extend the duration of follow-up.

In conclusion, insulinoma is the most common type of functional PNETs, with symptoms of loss of consciousness, sweating, vertigo and so on. The incidence of pancreatic fistula, bleeding and major complications after pancreatic head/neck insulinoma was higher than that after pancreatic body/tail insulinoma. It suggests to alert above complications after the operation of pancreatic head/neck insulinoma. The intraoperative suture of high-risk vessels prone to bleeding should be performed if necessary.

## CRediT authorship contribution statement

**Wei Li:** Writing – review & editing, Writing – original draft, Visualization, Validation, Resources, Project administration, Methodology, Investigation, Funding acquisition, Formal analysis, Data curation, Conceptualization. **Yali Cheng:** Writing – review & editing, Resources, Methodology, Investigation, Data curation. **Qingyong Ma:** Writing – review & editing, Supervision, Resources, Project administration, Conceptualization. **Zheng Wu:** Writing – review & editing, Supervision, Software, Resources, Investigation, Data curation, Conceptualization. **Zheng Wang:** Writing – review & editing, Supervision, Software, Project administration, Methodology, Investigation, Data curation, Conceptualization.

## Data availability

The datasets used and/or analyzed during the current study are available from the corresponding author on reasonable request.

## Funding resources

The present study was supported by grants from Institutional Foundation of the 10.13039/100031934First Affiliated Hospital of Xi'an Jiaotong University (grant no. 2022MS-35, 2021HL-21 and 2021HL-42).

## Declaration of Competing Interest

The authors declare that they have no known competing financial interests or personal relationships that could have appeared to influence the work reported in this paper.
